# Clinical, Radiological and Histopathological Evaluation of Primary Colon Lymphoma

**DOI:** 10.34172/aim.2023.56

**Published:** 2023-07-01

**Authors:** Deniz Tazeoglu, Ahmet Cem Esmer, Tahsin Colak

**Affiliations:** ^1^Department of General Surgery, Faculty of Medicine Mersin University, Mersin, Turkey

**Keywords:** Colon, Diffuse large B-cell lymphoma, Lymphoma

## Abstract

**Background:**

In this study, we aimed to examine the clinical, radiological, histopathological, immunohistochemical, and prognostic features of a case series undergoing surgery for non-Hodgkin’s primary colon lymphoma (NHL).

**Methods:**

The data of six patients diagnosed with NHL who were operated on in our clinic between January 2010 and January 2020 were retrospectively analyzed. NHL was detected in six of the patients operated on for colon tumors. B (n=5) and T (n=1) cell lymphomas were detected based on their cellular subtypes.

**Results:**

The median age at diagnosis was 66 (52-70). The most common complaints were abdominal pain, weight loss, nausea, and vomiting. One patient underwent emergency surgery, and five underwent elective surgery due to obstruction. While CT was used in all patients, Positron emission tomography–computed tomography (PET/CT) was taken only in patients who underwent elective surgery. The masses were localized in the cecum in two patients, in the right colon in two patients, and in the transverse colon and sigmoid colon in one patient each. All patients underwent mesocolic resection. The mean tumor size was 7.51±2.20 (4.5-11) cm. The median number of total lymph nodes was 33 (18-44), and the median number of metastatic lymph nodes was 15 (4-39).

**Conclusion:**

The overall and disease-free survival of the patient with T-cell lymphoma was shorter than that of patients with B-cell colon lymphoma. NHL is a rare disease. The cellular subtype effectively determines the survival time and prognosis of NHL.

## Introduction

 Non-Hodgkin’s primary colon lymphoma (NHL) constitutes 10%‒20% of gastrointestinal system lymphomas and 0.2%‒0.6% of primary malignancies.^1,2^ The most common region for extranodal lymphoma is the stomach, followed by the ileum, cecum, and colon. NHL is an extranodal lymphoma of the colon originating from tissues other than the lymph nodes and areas that do not contain lymphoid tissue. Mucosal-associated lymphoid tissue (MALT) lymphoma and diffuse large B-cell lymphoma (DLBCL) are the most common types of gastrointestinal malignancies.^3^

 Dawson’s criteria differentiate NHL from systemic lymphoma with secondary intestinal involvement. Dawson’s criteria have five components: absence of peripheral lymphadenopathy when the disease is detected, absence of mediastinal lymph node enlargement, normal white blood cell count and standard bone marrow biopsy, and absence of liver and spleen involvement.^4^

 Symptoms of NHL vary according to the location of the involvement in the colon. Clinical manifestations include abdominal pain, palpable mass, obstruction, loss of appetite, nausea, vomiting, weight loss, hematochezia, changes in bowel habits, and perforation.^5^

 Management is controversial due to the rarity of NHL; therefore, optimal treatment has not been defined. Current treatment modalities are derived from case series and expert opinions rather than sizeable randomized control trials. Radical surgical resection followed by chemotherapy is the most commonly preferred treatment for NHL. It has been observed that better local disease control and more prolonged survival are achieved with this treatment method.^6^

## Materials and Methods

 The data of patients who were operated on for colon tumors in our clinic between January 2010 and January 2020 and who were found to have colon lymphoma were analyzed retrospectively.

 We analyzed 407 patients who underwent elective or emergency segmental or mesocolic colon resection due to colon tumors. A case series of 6 (1.5%) patients who were diagnosed with NHL as a result of histopathological examination and met Dawson’s criteria were included in the study. Patients with evidence of extraperitoneal disease, and leukemic or lymphomatous abnormalities in the blood were excluded from the study, and patients with disease only confined to the colon were included in the study. A patient diagnosed with colon lymphoma, mediastinal lymph node with pathological size and features, and lymphoma involvement in a different area together with the colon was excluded from the study.

 Demographic data (age, gender), preoperative endoscopy reports, operative type (elective – emergency), histopathological and immunohistochemical examination, postoperative mortality and morbidity, and overall and disease-free survival time of all patients included in the study were recorded.

 The study was presented to Mersin University Clinical Research Ethics Committee, and ethics committee approval was obtained for the study. Statistical Package for Social Sciences (SPSS) for Windows 22.0 was used for statistical analysis. Descriptive statistical methods (mean, standard deviation) were used to evaluate the data.

## Results

 A pathology of lymphoma was detected in 7 of 407 patients who underwent emergency or elective surgery for colon tumors. One patient was excluded from the study because enlargement was detected in the mediastinal lymph node and the colon, and he did not meet Dawson’s criteria.

 Of the patients, 2 (33%) were female, and 4 (67%) were male. The median age was 62 (52‒70) years. There was a history of comorbid diseases in four patients. The patients had no known history of malignancy prior to NHL. None of the patients had a history of immunosuppressive therapy.

 The patients’ symptoms on admission to the hospital were: abdominal pain, nausea/vomiting, constipation, diarrhea, and weight loss. Examination findings at the time of admission to the hospital included abdominal tenderness, palpable mass, distention, and intestinal obstruction.

 The leukocyte count and distribution were normal in complete blood count in all patients. When biochemical parameters were examined, lactate dehydrogenase and total bilirubin were high in all patients. Coagulation values were normal. Emergency surgery (1) was performed in one patient due to colonic obstruction, and elective surgery (5) was performed in the other patients.

 Colonoscopy was performed in five patients (83%) who underwent elective surgery. The location of the colon lymphoma, the characteristics of the mass, and histopathological diagnosis were determined by colonoscopy. According to the postoperative histopathological examination, B-cell lymphoma was detected in five patients, and T-cell lymphoma was detected in one patient. As an endoscopic finding, a polypoid growing mass extending towards the lumen was detected in all of the patients who underwent endoscopy, while ulcerated vascular areas were found in the middle of the lesion in T-cell colon lymphoma, unlike the others.

 All patients underwent computed tomography of the abdomen. Positron emission tomography–computed tomography (PET/CT) was performed in five (83%) of the patients, and it was determined that there was no FDG uptake, except for the mass in the colon. Magnetic resonance imaging (MRI) was not used in any patients.

 All patients underwent mesocolic excision based on the location of the colon lymphoma during the surgery. The mean tumor size was 7.51 ± 2.20 (4.5‒11) cm. The median number of total lymph nodes removed during resection was 33 (18‒44). As for metastatic lymph nodes, the median number of the patients was 15 (4‒39). Distant metastases were absent in all patients. All patients were Stage IE according to the Ann Arbor staging system (Table 1).

**Table 1:** Postoperative Follow-up and Pathology Results of the Patients

**Patients**	**1st Patient**	**2nd Patient**	**3rd Patient**	**4th Patient**	**5th Patient**	**6th Patient**
Age	70	59	52	64	61	66
Gender	Male	Female	Male	Male	Female	Male
Cell type	B-cell	B-cell	B-cell	B-cell	B-cell	T-cell
Subtype	Diffuse large B-cell	Diffuse large B-cell	Extranodal marginal zone lymphoma	Diffuse large B-cell	Extranodal marginal zone lymphoma	Mature T-cell lymphoma
Total lymph nodes	18	44	33	29	42	22
Metastatic lymph node	15	39	27	18	5	4
Extracolon involvement	-	-	-	-	-	-
Stage (Ann Arbor)	IE	IE	IE	IE	IE	IE
Tumor location	Sigmoid colon	Cecum	Right colon	Right colon	Transfer colon	Cecum
Tumor size (cm)	7.2	11.1	8.5	7.5	6.3	4.5
Hospital stay (days)	13	11	7	9	6	15
Alive/Exitus	Alive	Exitus	Alive	Alive	Alive	Exitus
Disease-free survival (months)	89	41	102	83	71	11
Overall survival time (months)	89	56	102	83	71	33
Immunohistochemical examination						
CD3	-	+	+	-	-	+
CD10	-	-	-	-	-	+
CD20	+	+	+	+	+	-
CD23	-	+	+	-	-	-
CD43	+	+	-	+	+	-
Bcl 2	+	+	+	-	+	-
Cyclin D1	-	-	-	-	-	+
Ki 67	50%	60%	50%	40%	5%	80%

 Immunohistochemical examination was performed on all patients. As immunohistochemical examination, CD3, CD10, CD20, CD23, CD43, Bcl 2, cyclin D1, Ki67 were evaluated for all patients. All B-cell lymphoma patients had CD20 staining, 4 (80%) had CD43 and Bcl2, 2 (40%) had CD3 and CD23 staining, while none of the patients had CD10 and Cyclin D1 staining. Ki 67 was found between 5% and 60% in patients with B-cell lymphoma. The patient with T cell lymphoma was found to be CD3 + , CD10 + , CD20-, CD23-, CD43-, Bcl2-, Cyclin D1 + , and Ki 67 was found to be 80% (Table 1).

 All of the patients were followed up in the service after the operation, and there was no need for follow-up in the intensive care unit. All patients were discharged with recovery. The mean hospital stay was 10.2 ± 3.5 (6‒15) days.

 In the postoperative service follow-up, blood transfusion was performed due to wound infection in one patient and low hemoglobin in two patients. Therefore, according to the Clavien-Dindo classification, a 1^st^-degree complication was found in one patient, and a 2^nd^-degree complication was found in two patients. All patients were given adjuvant chemotherapy after resection in line with current information.

 While two patients died during the postoperative follow-up, four patients are alive, and the follow-up continues. Recurrence was detected in the abdomen of two patients who died. Recurrence was detected at 41 months in the patient with B-cell colon lymphoma and 11 months in the patient with T-cell colon lymphoma (Table 1).

## Discussion

 NHL is a rare disorder. In our study, NHL constituted 1.4% (6 out of 407 patients) of colonic malignancies, which is slightly above the 0.2%‒0.6% rate found in the literature.^1,7^ Since lymphomas in the gastrointestinal tract are rarely seen in the colon, the literature on colonic lymphomas is scarce. However, there are gastric and small bowel lymphomas in the current case series.^8,9^ Since the number of gastric and small bowel lymphomas is higher, the treatment principles are better determined.^10^

 While NHLs are most common between the 5th and 7th decades; the mean age at diagnosis is between 50 and 55.^7^ In our study, the mean age was 62 years. In addition, the patients had a male preponderance (M/F = 2/1), which is consistent with current studies.^2^

 The two most common symptoms in our patients were abdominal pain (67%) and weight loss (67%) in four patients each. It is correlated with those seen in the literature at rates of 40%‒90% and 27%‒80%, respectively.^11,12^ The most common site of NHL in the colon is the cecum, as most lymphoid tissue in the colon is located here.^13,14^ The cecum (33%) and right colon (33%) were the most frequently involved sites in our study, with two patients each.

 The most widely used staging system in clinical practice is the Lugano classification based on the Ann Arbor system modified by Carbone.^15^ The patients in our study had only one extranodal region involvement. Therefore, the stage of six patients was Stage IE. Five (83%) of the patients in our study had B-cell lymphoma, and this rate is consistent with the current literature. In addition, B cell marker CD 20 was reported as positive in all patients.

 According to the World Health Organization (WHO) classification, B-cell lymphomas are classified as DLBCL, extranodal marginal zone lymphoma, mucosa-associated lymphoid tissue (MALT) associated lymphoma, mantle cell lymphoma, Burkitt lymphoma, and follicular lymphoma.^16^ DLBCL is the most common histological subtype affecting the gastrointestinal tract and colon.^17^ DLBCL consists of rapidly proliferating cells and is more aggressive than other B-cell lymphomas. Among our study’s B-type NHL patients, three had DLBCL, and two had extranodal marginal zone lymphoma.

 In the current literature, NHLs are associated with post-transplant or immunosuppression-induced immune disorders.^18^ However, there was no immunosuppression in the patients in our study.

 NHL treatment requires a multidisciplinary approach. Combined with chemotherapy and surgery, radiotherapy may also be required in selected cases. The general approach in treatment is to administer adjuvant chemotherapy in select patients after surgical resection. Surgical resection provides important prognostic information; it enables us to understand the necessity of adjuvant chemotherapy, and prevents complications such as bleeding, obstruction, and perforation.^1,12,19^ The most commonly applied regimen as adjuvant chemotherapy is cyclophosphamide, doxorubicin, vincristine, and prednisone (CHOP), with a 5-year survival of 35%‒45%.^19,20^ Survival was further increased with the combination of CHOP treatment with rituximab (R-CHOP).^21,22^ Adjuvant radiotherapy can increase locoregional control in cases with incomplete resection. There are also case reports in the literature who went into remission with radiotherapy.^23,24^ It is recommended to be directed to clinical studies with aggressive stages III and IV.^25^

 All of the patients in our study underwent surgical treatment, and due to their early stage, chemotherapy was not considered necessary, and they were followed up in the outpatient clinic. Recurrence was detected in two patients during follow-up. Adjuvant chemotherapy (CHOP) regimen was given to patients with recurrence, and disease progression continued. Two patients died due to disease-related complications.

## Conclusion

 NHL is a rare disease, primarily affecting male patients between 5 and 7 decades. It most often involves the cecum in the colon. The most common reasons for admission are abdominal pain and weight loss. Histologically, it is usually B-cell type. Therefore, its treatment requires a multidisciplinary approach. Five-year survival ranges from 33-58%.
